# Comparative Analysis of OHIP Versions for Research on Temporomandibular Disorders

**DOI:** 10.1111/joor.70075

**Published:** 2025-10-07

**Authors:** Adrian Ujin Yap, Ni Luh Dewi, Emelia Syafiera, Carolina Marpaung

**Affiliations:** ^1^ Division of Dentistry Ng Teng Fong General Hospital and Faculty of Dentistry, National University Health System Singapore Singapore; ^2^ National Dental Research Institute Singapore, National Dental Centre Singapore and Duke‐NUS Medical School, Singapore Health Services Singapore Singapore; ^3^ Department of Prosthodontics, Faculty of Dentistry Universitas Trisakti Jakarta Indonesia; ^4^ Department of Orofacial Pain and Dysfunction Academic Centre for Dentistry Amsterdam (ACTA), University of Amsterdam and Vrije Universiteit Amsterdam Amsterdam the Netherlands

**Keywords:** correlation analysis, dimensions, domains, oral health, quality of life, temporomandibular disorders

## Abstract

**Background:**

An international team has recommended assessing oral health‐related quality of life (OHRQoL) in four dimensions and using the Oral Health Impact Profile‐5 (OHIP‐5) for all oral health conditions.

**Objectives:**

This study compared OHIP summary, domain, and dimension scores among individuals with no and different Temporomandibular Disorder (TMD) symptoms, examined correlations across OHIP versions, and explored the use of OHIP‐5 for TMD research.

**Methods:**

Young adults completed the Five TMD Symptoms (5Ts) screener and items from the OHIP‐49, OHIP‐14, OHIP‐5, and OHIP‐TMD. Participants were categorised into no (NT), intra‐articular (IT), pain‐related (PT), and combined (CT) TMD symptom groups. Data were analysed with the chi‐square test and nonparametric tests, including Spearman's correlation (*α* = 0.05).

**Results:**

The final sample consisted of 573 individuals (mean age 19.3 years [SD 1.3]; 82.6% women), of whom 64.0% reported NT, while 21.8%, 4.2%, and 10.0% had IT, PT, and CT symptoms, respectively. Significant differences in summary scores were consistent across participant groups (CT/PT/IT>NT; CT>IT) for all OHIP versions, though variations were observed in domain and dimension scores. Notably, no significant differences in the Psychosocial Impact dimension were identified for the OHIP‐5. Summary scores for the OHIP‐49 and OHIP‐14 were very strongly correlated with those for OHIP‐TMD, whereas the OHIP‐5 demonstrated a strong correlation (*r*_*s*_ = 0.86–0.94).

**Conclusions:**

A four‐dimensional impact framework is suitable for use in TMD research. While the OHIP‐5 can evaluate overall OHRQoL, its substitution for OHIP‐14 or OHIP‐TMD should be considered only when brevity is prioritised over dimension‐specific assessment.

## Graphical abstract

OHIP versions show higher scores in painful TMDs and strong correlations with OHIP‐TMD, supporting the OHIP‐5 for overall OHRQoL assessment.

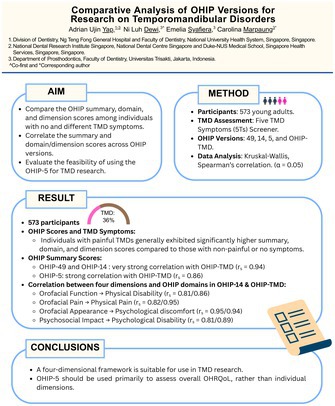

## 1 Background

Temporomandibular Disorders (TMDs) are a diverse group of musculoskeletal conditions affecting the temporomandibular joints (TMJs), jaw muscles, and related structures [1]. They represent a major public health concern, with a global prevalence of approximately 34%, and are often associated with chronic overlapping pain conditions such as fibromyalgia and irritable bowel syndrome [1, 2]. Women, particularly those aged 20–40, are especially vulnerable to TMDs [3, 4]. In addition to gender, sex hormones, and age, other biopsychosocial factors contributing to TMDs include genetics, trauma, oral behaviours, sleep disorders, somatic symptoms, and psychological distress [4, 5, 6]. The primary symptoms of TMDs consist of TMJ and jaw muscle pain, headaches, TMJ noises, and both closed and open jaw locking [7, 8]. The Diagnostic Criteria for TMDs (DC/TMD), along with its stratified reporting system, classifies TMD conditions and their symptoms into three categories: intra‐articular (IT), pain‐related (PT), and combined (CT) [8, 9]. TMDs have been found to significantly diminish oral health‐related quality of life (OHRQoL) [10, 11, 12]. In contrast, therapeutic interventions, including psychological and occlusal appliance therapy, were shown to improve the OHRQoL of individuals suffering from TMDs [13].

OHRQoL is a complex construct that assesses the impact of oral health and related conditions on an individual's overall well‐being and daily functioning [13, 14, 15]. As a dental patient‐reported outcome measure (dPROM), OHRQoL focuses on assessing patients' subjective experiences, which is essential for evaluating treatment efficacy from patients' perspectives and is necessary for evidence and value‐based care [13, 15]. Among the various measures of OHRQoL, the 14‐item short form of the Oral Health Impact Profile (OHIP‐14) is commonly employed in TMD research [10, 11, 12, 16, 17]. However, as a generic measure, the OHIP does not specifically address symptoms and effects related to TMDs and is prone to higher floor effects, as some of the surveyed items may be less common or applicable to TMDs [14, 18]. To overcome these limitations, Durham et al. [19, 20] developed the OHIP‐TMD, a condition‐specific OHRQoL instrument that includes 22 items, 20 of which are derived from the original 49‐item OHIP and 2 added from qualitative research with TMD patients.

Founded on Locker's conceptual model of oral health, the OHIP‐49, OHIP‐14, and OHIP‐TMD each consist of seven domains: Functional Limitation (Do1), Physical Pain (Do2), Psychological Discomfort (Do3), Physical Disability (Do4), Psychological Disability (Do5), Social Disability (Do6), and Handicap (Do7) [21]. However, empirical data did not support the OHIP's seven‐domain structure, and exploratory factor analysis revealed four distinct dimensions: Oral Function (Di1), Orofacial Pain (Di2), Orofacial Appearance (Di3), and Psychosocial Impact (Di4) [15, 22, 23]. The four‐dimensional framework was subsequently confirmed and validated through further analyses, leading to a more psychometrically sound and clinically plausible structure for both the OHIP and OHRQoL as a whole [15, 24]. Based on these new insights, an international team of oral health researchers proposed using the four dimensions as a standard metric to assess the impact of oral conditions and the outcomes of dental interventions, which required re‐mapping and scoring of various OHIP versions [15, 25]. Additionally, the four‐dimensional framework has been adopted to assess OHRQoL in orofacial pain conditions, including TMDs [26]. Given the strong correlations observed among the summary scores for the 49‐, 14‐, and 5‐item OHIP versions, and the fact that the OHIP‐5 explained 90% of the variance in OHIP‐49 scores, the OHIP‐5 was recommended for measuring OHRQoL in all new studies using the OHIP, regardless of settings and oral health conditions [15, 27].

In light of the context above, the objectives of this study were to: (1) compare the summary, domain, and dimension OHIP scores among individuals with no and different TMD symptoms, (2) correlate the summary and domain/dimension OHIP scores across generic and TMD‐specific OHIP measures, and (3) evaluate the feasibility of using the OHIP‐5 in place of the OHIP‐14 and OHIP‐TMD for TMD research. The research hypotheses were: (a) Individuals with painful TMDs (PT and CT) exhibit significantly higher summary and domain/dimension scores than those with non‐painful TMDs (IT) or no TMD (NT) symptoms, (b) The OHIP‐49, OHIP‐14, OHIP‐TMD, and OHIP‐5 are strongly correlated, and (c) The OHIP‐5 demonstrates measurement coherence with OHIP‐14 and OHIP‐TMD, supporting its potential utility for assessing OHRQoL in individuals with TMDs.

## 2 Methods

### 2.1 Study Participants

This cross‐sectional observational study received ethical approval from the Institutional Review Board of the Faculty of Dentistry, Universitas Trisakti (ID: 031/S3/KEPK/FKG/6/2023). A sample size of at least 112 participants across four comparison groups was required to achieve 95% statistical power with a significance level of 0.05. This calculation was conducted using G*Power software (Version 3.1.9.3) and an ANOVA model, based on an effect size of 0.40 for OHRQoL, as informed by previous research [28, 29]. Young adults were recruited using a non‐probabilistic voluntary sampling method at Indonesia's largest private university. Potential participants were engaged through intranet announcements, personal connections, and face‐to‐face interactions. The inclusion criteria were age 18–24 years and proficiency in English, while the exclusion criteria included a history of orofacial trauma or orthognathic surgery, ongoing care for debilitating psychological or physical conditions, and incomplete surveys. Potential participants were provided with the study details, and informed consent was obtained before completing an online survey that encompassed demographic information, the Five TMD Symptoms (5Ts) screener, and items from the various OHIP versions [16, 17, 19, 20, 30].

### 2.2 Study Measures

#### 2.2.1 TMD Symptoms

The 5Ts screener, derived from the DC/TMD Symptom Questionnaire (SQ), was used to evaluate five primary TMD symptoms: orofacial pain, headaches, joint noises, closed locking, and open locking. It was benchmarked against the full DC/TMD diagnostic standard (including the complete SQ, clinical examination, supplementary imaging, and diagnostic algorithms) and demonstrated excellent diagnostic accuracy for identifying all TMDs, as well as pain‐related and intra‐articular conditions, with area under the receiver operating characteristic curve (AUC) values of ≥ 0.98, sensitivity > 96%, and specificity of 100% [30]. While the 5Ts effectively identifies individuals with TMDs, it serves as a screening tool rather than a diagnostic replacement. Dichotomous ‘no’ and ‘yes’ responses were employed to record the presence of TMD symptoms over the past 30 days. Participants were classified as ‘no TMD symptoms’ (NT) if they answered ‘no’ to all items, as IT if they answered ‘yes’ to any of the three intra‐articular items, as PT if they answered ‘yes’ to either of the two pain‐related items, and as CT if they answered ‘yes’ to both IT and PT items.

#### 2.2.2 OHIP Versions

The OHIP‐49, OHIP‐14, OHIP‐5, and OHIP‐TMD, comprising forty‐nine, fourteen, five, and twenty‐two items, were used to assess OHRQoL across seven domains and four dimensions [16, 17, 19, 20]. The item distributions for each OHIP version, together with those utilised to compute domain and dimension scores, are listed in Table 1 [15, 25]. While domain scores do not apply to the OHIP‐5, the two additional items from the OHIP‐TMD, specifically ‘difficulty in mouth opening and closing’ and ‘pain while talking,’ were incorporated into Di1 (Oral Function) and Di2 (Orofacial Pain) following their relevant domains. Items were scored using a 5‐point response scale, with ‘never’ assigned 0 points, ‘hardly’ 1 point, ‘occasionally’ 2 points, ‘fairly often’ 3 points, and ‘very often’ 4 points. Summary, domain, and dimension OHIP scores were calculated by summing the ordinal values of all items from the different OHIP versions, as well as domain‐ and dimension‐specific items. Given the limited relevance of ‘flavor in food’ to TMDs, item 26 was excluded from the secondary OHIP‐5 analysis. Higher summary OHIP scores indicate poorer overall OHRQoL, whereas higher domain and dimension scores reflect more focused impairments within these areas.

**TABLE 1:** Items and domain/dimension components of the OHIP‐49, OHIP‐14, OHIP‐5, and OHIP‐TMDs.

Item	Description	OHIP‐49	OHIP‐14	OHIP‐5	OHIP‐TMD
1.	Difficulty chewing	Do1; Di1		Do1; Di1	1Do1, Di1
2.	Trouble pronouncing words	Do1; Di1	Do1; Di1		
3.	Noticed tooth which doesn't look right	Do1; Di3			
4.	Appearance affected	Do1; Di3			
5.	Breath stale	Do1; −			
6.	Taste worse	Do1; Di1	Do1; Di1		
7.	Food catching	Do1; −			
8.	Digestion worse	Do1; −			
9.	Dentures not fitting	Do1; −			
10.	Painful aching	Do2; Di2	Do2; Di2	Do2; Di2	Do2; Di2
11.	Sore jaw	Do2; Di2			Do2; Di2
12.	Headache	Do2; Di2			Do2; Di2
13.	Sensitive teeth	Do2; Di2			
14.	Toothache	Do2; Di2			
15.	Painful gum	Do2; Di2			
16.	Uncomfortable to eat	Do2; Di1	Do2; Di1		Do2; Di1
17.	Sore spots	Do2; Di2			
18.	Uncomfortable dentures	Do2; −			
19.	Worried	Do3; Di3			Do3; Di3
20.	Self conscious	Do3; Di3	Do3; Di3		Do3; Di3
21.	Miserable	Do3; −			Do3; —
22.	Uncomfortable about appearance	Do3; Di3		Do3; Di3	
23.	Tense	Do3; Di4	Do3; Di4		Do3; Di4
24.	Speech unclear	Do4; Di1			
25.	Others misunderstood	Do4; Di1			
26.	Less flavour in food	Do4; Di1		Do4; Di1	
27.	Unable to brush teeth	Do4; −			
28.	Avoid eating	Do4; Di1			Do4; Di1
29.	Diet unsatisfactory	Do4; Di1	Do4; Di1		
30.	Unable to eat with dentures	Do4; −			
31.	Avoid smiling	Do4; Di3			
32.	Interrupt meals	Do4; Di1	Do4; Di1		Do4; Di1
33.	Sleep interrupted	Do5; Di4			Do5; Di4
34.	Upset	Do5; Di4			Do5; Di4
35.	Difficult to relax	Do5; Di4	Do5; Di4		Do5; Di4
36.	Depressed	Do5; Di4			Do5; Di4
37.	Concentration affected	Do5; Di4			Do5; Di4
38.	Been embarrassed	Do5; Di4	Do5; Di4		
39.	Avoid going out	Do6; Di4			
40.	Less tolerant of others	Do6; Di4			
41.	Trouble getting on with others	Do6; Di4			
42.	Irritable with others	Do6; Di4	Do6; Di4		Do6; Di4
43.	Difficulty doing jobs	Do6; Di4	Do6; Di4	Do6; Di4	Do6; Di4
44.	Health worsened	Do7; Di4			
45.	Financial loss	Do7; Di4			
46.	Unable to enjoy people's company	Do7; Di4			
47.	Life unsatisfying	Do7; Di4	Do7; Di4		Do7; Di4
48.	Unable to function	Do7; Di4	Do7; Di4		
49.	Unable to work	Do7; Di4			Do7; Di4
50.	Difficulties in mouth opening and closing				Do1; Di1
51.	Pain while talking				Do2; Di2

Abbreviations: Di1, Orofacial function; Di2, Orofacial pain; Di3, Orofacial appearance; Di4, Psychosocial impact; Do1, Functional limitation; Do2, Physical pain; Do3, Psychological discomfort; Do4, Physical disability; Do5, Psychological disability; Do6, Social disability; Do7, Handicap.

### 2.3 Statistical Analyses

All statistical analyses were performed using version 27.0 of the Statistical Package for the Social Sciences (SPSS) software (IBM Corporation, Armonk, New York, USA), with the significance level set at 0.05. Qualitative data were expressed as frequencies with percentages and analysed using the chi‐square test with Bonferroni correction. Quantitative data were reported as means with standard deviations (SDs), accompanied by medians with interquartile ranges (IQRs), and evaluated for normality using the Shapiro–Wilk test. Due to nonnormal distributions, quantitative data were analysed using the Kruskal–Wallis and post hoc Mann–Whitney *U* tests, as well as Spearman's rank‐order correlations to assess associations between summary, domain, and dimension scores. Correlation coefficients (*r*_*s*_) of 0.1, 0.4, 0.7, and 0.9 were used as thresholds to define weak, moderate, strong, and very strong associations, correspondingly [31].

## 3 Results

Of the 587 eligible young adults who volunteered for the study, 14 were excluded because of incomplete surveys. The final sample consisted of 573 participants, 82.6% of whom were women, with a mean age of 19.3 years (SD = 1.3). Among the study participants, 64.0% reported no TMD symptoms, while 21.8%, 4.2%, and 10.0% had IT, PT, and CT symptoms, respectively. Table 2 shows the summary scores for the four different OHIP versions. Significant differences in summary OHIP scores were observed across the participant groups for all four OHIP versions (CT, PT, IT>NT; CT>IT).

**TABLE 2:** Demographics and summary OHIP scores for the four participant groups.

Variables		All participants	No TMD (NT)	Intra‐articular TMD (IT)	Pain‐related TMD (PT)	Combined TMD (CT)	*p*, post hoc
Total	*n* (%)	573 (100)	367 (64.0)	125 (21.8)	24 (4.2)	57 (10.0)	
Age	Mean (SD)	19.33 (1.25)	19.30 (1.23)	19.36 (1.34)	19.33 (1.43)	19.44 (1.13)	0.790^*^
	Median (IQR)	19.00 (2.00)	19.00 (2.00)	19.00 (2.00)	19.00 (3.00)	19.00 (1.00)	
Sex	Female, *n* (%)	473 (82.6)	292 (79.6)	110 (88.0)	22 (91.7)	49 (86)	0.082^^^
	Male, *n* (%)	100 (17.4)	75 (20.4)	15 (12.0)	2 (8.3)	8 (14)	
Summary scores							
OHIP‐49	Mean (SD)	17.31 (18.00)	14.47 (16.09)	18.01 (16.34)	22.88 (19.23)	31.75 (24.36)	< 0.001^*^ CT,PT,IT>NT CT>IT
	Median (IQR)	12.00 (20.00)	10.00 (18.00)	13 (18.50)	21.50 (28.75)	26.00 (31.50)	
OHIP‐14	Mean (SD)	4.30 (5.41)	3.55 (4.77)	4.26 (4.75)	5.79 (5.85)	8.56 (7.93)	< 0.001^*^ CT,PT,IT>NT CT>IT
	Median (IQR)	2.00 (6.00)	2.00 (5.00)	3.00 (5.00)	4.00 (6.50)	6.00 (11.50)	
OHIP‐5	Mean (SD)	1.88 (2.23)	1.52 (1.96)	2.02 (2.16)	2.50 (2.62)	3.65 (2.88)	< 0.001^*^ CT,PT,IT>NT CT>IT
	Median (IQR)	1.00 (3.00)	1.00 (2.00)	1.00 (3.00)	2.00 (3.00)	3.00 (4.00)	
OHIP‐TMD	Mean (SD)	7.26 (8.62)	5.62 (7.07)	7.62 (7.99)	10.33 (8.70)	15.68 (12.88)	< 0.001^*^ CT,PT,IT>NT CT>IT
	Median (IQR)	4.00 (9.00)	3.00 (8.00)	5.00 (10.00)	7.50 (11.50)	12.00 (17.00)	

*Note:* Results of ^chi‐square test with Bonferroni correction and *Kruskal–Wallis and Mann–Whitney *U* tests. Bold indicates *p* < 0.05.

Abbreviations: IQR, interquartile range; SD, standard deviation.

Tables 3 and 4 present the domain and dimension scores for the various OHIP versions across the four participant groups. Significant differences were noted in the following domain scores:

- Functional Limitation (Do1): For OHIP‐49 and OHIP‐TMD, CT>IT, PT, NT; IT>NT. For OHIP‐14, CT>IT, NT.
- Physical Pain (Do2): For OHIP‐49, OHIP‐14, and OHIP‐TMD, CT, PT, IT>NT; CT>IT.
- Psychological Discomfort (Do3): For OHIP‐49 and OHIP‐TMD, CT, PT>NT; CT>IT. For OHIP‐14, CT, PT, IT>NT; CT>IT.
- Physical Disability (Do4): For OHIP‐49 and OHIP‐14, CT>IT, NT. For OHIP‐TMD, CT>PT, IT, NT.
- Psychological Disability (Do5): For OHIP‐49 and OHIP‐14, CT, PT>NT; CT>IT. For OHIP‐TMD, CT, PT>IT, NT.
- Social Disability (Do6): For OHIP‐49, OHIP‐14, and OHIP‐TMD, CT>IT, NT.
- Handicap (Do7): For OHIP‐49, OHIP‐14, and OHIP‐TMD, CT>IT, NT.

**TABLE 3:** OHIP domain scores for the four participant groups.

Variables		All participants	No TMD (NT)	Intra‐articular TMD (IT)	Pain‐related TMD (PT)	Combined TMD (CT)	p*, post hoc
Functional limitation (Do1)							
OHIP‐49	Mean (SD)	4.02 (4.14)	3.40 (3.70)	4.38 (4.15)	4.42 (4.51)	7.02 (5.26)	**< 0.001** CT>IT,PT,NT IT>NT
	Median (IQR)	3.00 (6.00)	2.00 (6.00)	3.00 (5.00)	3.50 (6.00)	7.00 (8.00)	
OHIP‐14	Mean (SD)	0.42 (0.83)	0.33 (0.70)	0.45 (0.87)	0.54 (1.06)	0.89 (1.21)	**< 0.001** CT>IT,NT
	Median (IQR)	0.91 (1.00)	0.00 (0.00)	0.00 (1.00)	0.00 (1.00)	0.00 (2.00)	
OHIP‐TMD	Mean (SD)	0.40 (0.91)	0.16 (0.46)	0.58 (0.96)	0.50 (0.98)	1.54 (1.69)	**< 0.001** CT>IT,PT,NT IT>NT
	Median (IQR)	0.00 (0.00)	0.00 (0.00)	0.00 (1.00)	0.00 (1.00)	1.00 (2.50)	
Physical pain (Do2)							
OHIP‐49	Mean (SD)	4.12 (4.00)	3.29 (3.47)	4.56 (3.57)	6.29 (4.74)	7.61 (5.30)	**< 0.001** CT,PT,IT>NT CT>IT
	Median (IQR)	3.00 (5.00)	2.00 (5.00)	4.00 (5.00)	6.00 (6.50)	7.00 (5.50)	
OHIP‐14	Mean (SD)	0.81 (1.09)	0.65 (0.93)	0.88 (1.07)	1.33 (1.46)	1.47 (1.50)	**< 0.001** CT,PT,IT>NT CT>IT
	Median (IQR)	0.00 (1.00)	0.00 (1.00)	1.00 (2.00)	1.00 (2.00)	1.00 (2.00)	
OHIP‐TMD	Mean (SD)	1.57 (2.17)	1.08 (1.60)	1.68 (1.90)	2.79 (2.83)	3.95 (3.46)	**< 0.001** CT,PT,IT>NT CT>IT
	Median (IQR)	1.00 (2.00)	0.00 (2.00)	1.00 (3.00)	2.00 (4.50)	3.00 (4.50)	
Psychological discomfort (Do3)							
OHIP‐49	Mean (SD)	3.32 (3.60)	2.84 (3.26)	3.57 (3.76)	4.50 (3.53)	5.40 (4.35)	**< 0.001** CT,PT>NT CT>IT
	Median (IQR)	2.00 (5.00)	2.00 (5.00)	3.00 (5.00)	5.00 (6.25)	5.00 (7.50)	
OHIP‐14	Mean (SD)	1.18 (1.48)	0.97 (1.33)	1.26 (1.53)	1.63 (1.41)	2.18 (1.85)	**< 0.001** CT,PT,IT>NT CT>IT
	Median (IQR)	1.00 (2.00)	0.00 (2.00)	1.00 (2.00)	2.00 (2.75)	2.00 (4.00)	
OHIP‐TMD	Mean (SD)	2.68 (2.93)	2.29 (2.62)	2.84 (3.06)	3.83 (3.02)	4.37 (3.72)	**< 0.001** CT,PT>NT CT>IT
	Median (IQR)	2.00 (4.00)	1.00 (4.00)	2.00 (4.00)	4.00 (4.50)	3.00 (6.00)	
Physical disability (Do4)							
OHIP‐49	Mean (SD)	2.66 (4.01)	2.32 (3.79)	2.49 (3.51)	3.54 (4.28)	4.84 (5.46)	**< 0.001** CT>IT,NT
	Median (IQR)	1.00 (4.00)	0.00 (3.00)	1.00 (4.00)	2.00 (5.75)	4.00 (7.50)	
OHIP‐14	Mean (SD)	0.66 (1.22)	0.57 (1.10)	0.58 (0.96)	0.83 (1.27)	1.37 (1.97)	**0.013** CT>IT,NT
OHIP‐TMD	Mean (SD)	0.79 (1.29)	0.67 (1.15)	0.79 (1.13)	0.71 (1.23)	1.63 (1.99)	**0.001** CT>PT,IT,NT
	Median (IQR)	0.00 (1.00)	0.00 (1.00)	0.00 (1.50)	0.00 (1.75)	1.00 (2.50)	
Psychological disability (Do5)							
OHIP‐49	Mean (SD)	1.54 (2.61)	1.19 (2.19)	1.55 (2.69)	2.17 (2.41)	3.53 (3.85)	**< 0.001** CT,PT>NT CT>IT
	Median (IQR)	0.00 (2.00)	0.00 (1.00)	0.00 (2.00)	1.50 (4.50)	2.00 (5.00)	
OHIP‐14	Mean (SD)	0.52 (0.97)	0.42 (0.84)	0.50 (0.93)	0.75 (0.90)	1.11 (1.47)	**< 0.001** CT,PT>NT CT>IT
	Median (IQR)	0.00 (1.00)	0.00 (1.00)	0.00 (1.00)	0.00 (2.00)	1.00 (2.00)	
OHIP‐TMD	Mean (SD)	1.20 (2.17)	0.89 (1.78)	1.26 (2.32)	1.83 (2.01)	2.84 (3.19)	**< 0.001** CT,PT>IT,NT
	Median (IQR)	0.00 (2.00)	0.00 (1.00)	0.00 (1.00)	1.00 (3.75)	2.00 (4.00)	
Social disability (Do6)							
OHIP‐49	Mean (SD)	0.60 (1.74)	0.54 (1.80)	0.43 (1.23)	0.71 (1.60)	1.25 (2.18)	**0.001** CT>IT,NT
	Median (IQR)	0.00 (0.00)	0.00 (0.00)	0.00 (0.00)	0.00 (0.00)	0.00 (2.00)	
OHIP‐14	Mean (SD)	0.28 (0.86)	0.26 (0.88)	0.18 (0.51)	0.29 (0.69)	0.61 (1.25)	**0.007** CT>IT,NT
	Median (IQR)	0.00 (0.00)	0.00 (0.00)	0.00 (0.00)	0.00 (0.00)	0.00 (1.00)	
OHIP‐TMD	Mean (SD)	0.28 (0.86)	0.26 (0.88)	0.18 (0.51)	0.29 (0.69)	0.61 (1.25)	**0.007** CT>IT,NT
	Median (IQR)	0.00 (0.00)	0.00 (0.00)	0.00 (0.00)	0.00 (0.00)	0.00 (1.00)	
Handicap (Do7)							
OHIP‐49	Mean (SD)	1.05 (2.22)	0.88 (2.13)	1.03 (1.94)	1.25 (1.92)	2.10 (3.08)	**< 0.001** CT>IT,NT
	Median (IQR)	0.00 (1.00)	0.00 (1.00)	0.00 (2.00)	0.00 (1.75)	1.00 (2.50)	
OHIP‐14	Mean (SD)	0.42 (0.96)	0.35 (0.86)	0.42 (0.91)	0.42 (0.72)	0.93 (1.49)	**< 0.001** CT>IT,NT
	Median (IQR)	0.00 (0.00)	0.00 (0.00)	0.00 (0.50)	0.00 (1.00)	0.00 (1.00)	
OHIP‐TMD	Mean (SD)	0.33 (0.79)	0.28 (0.74)	0.30 (0.62)	0.38 (0.65)	0.74 (1.26)	**0.003** CT>IT,NT
	Median (IQR)	0.00 (0.00)	0.00 (0.00)	0.00 (0.00)	0.00 (1.00)	0.00 (1.00)	

*Note:* Results of *Kruskal–Wallis and Mann–Whitney *U* tests. Bold indicates *p* < 0.05.

Abbreviations: IQR, interquartile range; NA, not applicable; SD, standard deviation.

**TABLE 4:** OHIP dimension scores for the four participant groups.

Variables		All participants	No TMD (NT)	Intra‐articular TMD (IT)	Pain‐related TMD (PT)	Combined TMD (CT)	p*, post hoc
Orofacial function (Di1)							
OHIP‐49	Mean (SD)	2.99 (4.25)	2.41 (3.68)	3.02 (3.80)	4.00 (5.79)	6.28 (6.02)	**< 0.001** CT>IT,PT,NT IT>NT
	Median (IQR)	1.00 (5.00)	1.00 (4.00)	2.00 (4.50)	1.50 (6.50)	5.00 (6.50)	
OHIP‐14	Mean (SD)	1.45 (2.16)	1.17 (1.84)	1.46 (1.90)	1.96 (2.74)	3.04 (3.37)	**< 0.001** CT>IT>NT
	Median (IQR)	0.00 (2.00)	0.00 (2.00)	1.00 (2.00)	1.00 (3.75)	2.00 (5.00)	
OHIP‐5	Mean (SD)	0.66 (1.08)	0.46 (0.85)	0.73 (1.12)	0.96 (1.33)	1.63 (1.52)	**< 0.001** CT>IT,PT>NT
	Median (IQR)	0.00 (1.00)	0.00 (1.00)	0.00 (1.00)	0.50 (1.75)	1.00 (2.50)	
OHIP‐TMD	Mean (SD)	1.56 (2.39)	1.09 (1.79)	1.80 (2.30)	1.79 (2.73)	3.95 (3.92)	**< 0.001** CT>IT,PT,NT IT>NT
	Median (IQR)	0.00 (2.00)	0.00 (2.00)	1.00 (3.00)	1.00 (2.75)	3.00 (4.50)	
Orofacial pain (Di2)							
OHIP‐49	Mean (SD)	3.71 (3.46)	2.97 (3.02)	4.10 (3.11)	5.63 (3.99)	6.79 (4.46)	**< 0.001** CT,PT,IT>NT CT>IT
	Median (IQR)	3.00 (5.00)	2.00 (5.00)	4.00 (4.00)	6.00 (5.75)	6.00 (5.50)	
OHIP‐14	Mean (SD)	0.45 (0.65)	0.39 (0.61)	0.46 (0.64)	0.75 (0.79)	0.70 (0.80)	**< 0.001** CT,PT>NT CT>IT
	Median (IQR)	0.00 (1.00)	0.00 (1.00)	0.00 (1.00)	1.00 (1.00)	1.00 (1.00)	
OHIP‐5	Mean (SD)	0.45 (0.65)	0.39 (0.61)	0.46 (0.64)	0.75 (0.79)	0.70 (0.80)	**0.003** CT,PT>NT CT>IT
	Median (IQR)	0.00 (1.00)	0.00 (1.00)	0.00 (1.00)	1.00 (1.00)	1.00 (1.00)	
OHIP‐TMD	Mean (SD)	1.20 (1.72)	0.81 (1.27)	1.26 (1.45)	2.21 (2.13)	3.18 (2.79)	**< 0.001** CT,PT>IT> NT
	Median (IQR)	0.00 (2.00)	0.00 (1.00)	1.00 (2.00)	2.00 (3.25)	2.00 (4.00)	
Orofacial appearance (Di3)							
OHIP‐49	Mean (SD)	4.12 (4.15)	3.50 (3.73)	4.53 (4.39)	4.88 (4.13)	6.91 (4.97)	**< 0.001** CT>IT>NT
	Median (IQR)	3.00 (6.00)	3.00 (6.00)	4.00 (5.50)	4.00 (6.75)	6.00 (7.00)	
OHIP‐14	Mean (SD)	0.86 (1.05)	0.70 (0.95)	0.94 (1.09)	1.21 (1.10)	1.53 (1.30)	**< 0.001** CT,PT,IT>NT CT>IT
	Median (IQR)	0.00 (2.00)	0.00 (1.00)	1.00 (2.00)	1.00 (2.00)	1.00 (3.00)	
OHIP‐5	Mean (SD)	0.64 (0.87)	0.55 (0.83)	0.73 (0.91)	0.67 (0.70)	1.04 (0.94)	**< 0.001** CT>IT>NT
	Median (IQR)	0.00 (1.00)	0.00 (1.00)	0.00 (1.00)	1.00 (1.00)	1.00 (2.00)	
OHIP‐TMD	Mean (SD)	1.60 (1.72)	1.34 (1.52)	1.74 (1.75)	2.21 (1.77)	2.70 (2.28)	**< 0.001** CT,PT,IT>NT CT>IT
	Median (IQR)	1.00 (3.00)	1.00 (2.00)	2.00 (3.00)	2.00 (3.75)	2.00 (3.00)	
Psychosocial impact (Di4)							
OHIP‐49	Mean (SD)	3.51 (6.14)	2.88 (5.80)	3.33 (5.40)	4.54 (5.32)	7.53 (8.35)	**< 0.001** CT,PT>NT CT>IT
	Median (IQR)	1.00 (4.00)	0.00 (3.00)	1.00 (4.00)	3.00 (7.00)	4.00 (10.50)	
OHIP‐14	Mean (SD)	1.54 (2.72)	1.29 (2.54)	1.41 (2.32)	1.88 (2.36)	3.30 (3.90)	**< 0.001** CT>IT,NT
	Median (IQR)	0.00 (2.00)	0.00 (2.00)	0.00 (2.00)	1.00 (3.00)	2.00 (5.00)	
OHIP‐5	Mean (SD)	0.13 (0.46)	0.12 (0.47)	0.10 (0.36)	0.13 (0.34)	0.28 (0.65)	0.056
	Median (IQR)	0.00 (0.00)	0.00 (0.00)	0.00 (0.00)	0.00 (0.00)	0.00 (0.00)	
OHIP‐TMD	Mean (SD)	2.14 (3.69)	1.70 (3.28)	2.04 (3.46)	2.92 (3.17)	4.84 (5.41)	**< 0.001** CT,PT>NT CT>IT
	Median (IQR)	0.00 (3.00)	0.00 (2.00)	0.00 (3.00)	2.50 (5.75)	3.00 (8.00)	

*Note:* Results of *Kruskal–Wallis and Mann–Whitney *U* tests. Bold indicates *p* < 0.05.

Abbreviations: IQR, interquartile range; SD, standard deviation.

Significant differences were discerned in the following dimension scores:

- Orofacial Function (Di1): For OHIP‐49 and OHIP‐TMD, CT>IT, PT, NT; IT>NT. For OHIP‐14, CT>IT> NT. For OHIP‐5, CT>IT, PT>NT.
- Orofacial Pain (Di2): For OHIP‐49, CT, PT, IT>NT; CT>IT. For OHIP‐14 and OHIP‐5, CT, PT>NT; CT>IT. For OHIP‐TMD, CT, PT>IT>NT.
- Orofacial Appearance (Di3): For OHIP‐49 and OHIP‐5, CT>IT>NT. For OHIP‐14 and OHIP‐TMD, CT, PT, IT>NT; CT>IT.
- Psychosocial Impact (Di4): For OHIP‐49 and OHIP‐TMD, CT, PT>NT; CT>IT. For OHIP‐14, CT>IT, NT. For OHIP‐5, no significant differences.

Table 5 presents the results of the correlation analysis among summary, domain, and dimension scores. The OHIP‐49 and OHIP‐14 summary scores exhibited very strong correlations with the OHIP‐TMD summary score (*r*_*s*_ = 0.94), while the OHIP‐5 summary score showed a strong association (*r*_*s*_ = 0.86). Similarly, the OHIP‐5 summary score was strongly correlated with the OHIP‐49 and OHIP‐14 summary scores (*r*_*s*_ = 0.84–0.85). For both OHIP‐14 and OHIP‐TMD, the dimensions of Orofacial Function (Di1), Orofacial Pain (Di2), Orofacial Appearance (Di3), and Psychosocial Impact (Di4) had the strongest correlations with the domains of Physical Disability (Do4; *r*_*s*_ = 0.81/0.86), Physical Pain (Do2; *r*_*s*_ = 0.82/0.95), Psychological Discomfort (Do3; *r*_*s*_ = 0.95/0.94), and Psychological Disability (Do5; *r*_*s*_ = 0.81/0.89), respectively. When the dimensions of OHIP‐5 were compared with the domains of OHIP‐14 and OHIP‐TMD, the following showed the strongest correlations:

- OHIP‐14: Orofacial Function (Di1) with Physical Disability (Do4; *r*_*s*_ = 0.66), Orofacial Function (Di1) minus item 26 with Physical Pain (Do2; *r*_*s*_ = 0.39), Orofacial Pain (Di2) with Physical Pain (Do2; *r*_*s*_ = 0.82), Orofacial Appearance (Di3) with Psychological Discomfort (Do3; *r*_*s*_ = 0.67), and Psychosocial Impact (Di4) with Social Disability (Do6: *r*_*s*_ = 0.81).
- OHIP‐TMD: Orofacial Function (Di1) with Functional Limitation (Do1; *r*_*s*_ = 0.68), Orofacial Function (Di1) minus item 26 with Functional Limitation (Do1; *r*_*s*_ = 0.88), Orofacial Pain (Di2) with Physical Pain (Do2; *r*_*s*_ = 0.73), Orofacial Appearance (Di3) with Psychological Discomfort (Do3; *r*_*s*_ = 0.69), and Psychosocial Impact (Di4) with Social Disability (Do6: *r*_*s*_ = 0.81).

**TABLE 5:** Correlations among summary, domain, and dimension scores.

Correlation variables					
Summary score	OHIP‐49	OHIP‐14	OHIP‐5	OHIP‐TMD	
OHIP‐49	1	0.93**	0.84**	0.94**	
OHIP‐14	0.93**	1	0.85**	0.94**	
OHIP‐5	0.84**	0.85**	1	0.86**	
OHIP‐TMD	0.94**	0.94**	0.86**	1	
**OHIP‐14**					
**Dimension**	**Orofacial function (Di1)**	**Orofacial pain (Di2)**	**Orofacial appearance (Di3)**	**Psychosocial impact (Di4)**	
Domain					
Summary score	0.84**	0.55**	0.74**	0.83**	
Functional limitation (Do1)	0.70**	0.24**	0.30**	0.40**	
Physical pain (Do2)	0.65**	0.82**	0.47**	0.50**	
Psychological discomfort (Do3)	0.57**	0.37**	0.95**	0.64**	
Physical disability (Do4)	0.81**	0.31**	0.42**	0.61**	
Psychological disability (Do5)	0.54**	0.29**	0.42**	0.81**	
Social disability (Do6)	0.42**	0.20**	0.25**	0.59**	
Handicap (Do7)	0.48**	0.26**	0.35**	0.72**	
**OHIP‐TMD**					
**Dimension**	**Orofacial function**	**Orofacial pain**	**Orofacial appearance**	**Psychosocial impact**	
Domain					
Summary score	0.79**	0.73**	0.82**	0.83**	
Functional limitation (Do1)	0.68**	0.49**	0.30**	0.45**	
Physical pain (Do2)	0.70**	0.95**	0.51**	0.59**	
Psychological discomfort (Do3)	0.53**	0.46**	0.94**	0.67**	
Physical disability (Do4)	0.86**	0.46**	0.48**	0.59**	
Psychological disability (Do5)	0.58**	0.55**	0.54**	0.89**	
Social disability (Do6)	0.41**	0.36**	0.32**	0.57**	
Handicap (Do7)	0.47**	0.37**	0.36**	0.64**	
**OHIP‐5 dimension**	**Orofacial function (Di1)**	**Orofacial function (Di1) minus item 26**	**Orofacial pain (Di2)**	**Orofacial appearance (Di3)**	**Psychosocial impact (Di4)**
OHIP‐14 domain					
Summary score	0.63**	0.41**	0.55**	0.70**	0.43**
Functional limitation (Do1)	0.37**	0.30**	0.24**	0.34**	0.19**
Physical pain (Do2)	0.52**	0.39**	0.82**	0.50**	0.25**
Psychological discomfort (Do3)	0.43**	0.28**	0.37**	0.67**	0.31**
Physical disability (Do4)	0.66**	0.35**	0.31**	0.51**	0.41**
Psychological disability (Do5)	0.44**	0.30**	0.29**	0.49**	0.41**
Social disability (Do6)	0.44**	0.28**	0.20**	0.39**	0.81**
Handicap (Do7)	0.41**	0.30**	0.26**	0.38**	0.41**
OHIP‐TMD domains					
Summary score	0.66**	0.47**	0.54**	0.71**	0.42**
Functional limitation (Do1)	0.68**	0.88**	0.31**	0.35**	0.31**
Physical pain (Do2)	0.56**	0.44**	0.73**	0.50**	0.32**
Psychological discomfort (Do3)	0.42**	0.27**	0.37**	0.69**	0.31**
Physical disability (Do4)	0.64**	0.37**	0.30**	0.55**	0.35**
Psychological disability (Do5)	0.57**	0.40**	0.42**	0.56**	0.41**
Social disability (Do6)	0.44**	0.28**	0.20**	0.39**	0.81**
Handicap (Do7)	0.41**	0.28**	0.24**	0.34**	0.44**

*Note:* Item 6: Taste worse (less flavour in food). Results of Spearman's correlation.

* Significant correlation.

** *p* < 0.01.

## 4 Discussion

This study compared OHIP summary, domain, and dimension scores among individuals with no and different TMD symptoms, and examined correlations across various OHIP versions. It is one of the first studies to evaluate the feasibility of using OHIP‐5 in place of the OHIP‐14 and OHIP‐TMD, which are widely applied in TMD research [10, 11, 12, 32]. Although the OHIP‐5 is commonly used in general population studies, its suitability for TMD research remains unestablished [33, 34]. A key concern is the limited item set, which includes content not directly pertinent to TMDs. Item 26 (‘less flavor in food’) accounts for one‐fifth of the OHIP‐5, yet bears minimal relevance to TMD symptomatology, and was excluded in the secondary analysis to evaluate whether its omission enhanced correlations with the OHIP‐14 and OHIP‐TMD. The first research hypothesis was confirmed, as individuals with painful TMDs generally exhibited significantly higher summary, domain, and dimension scores compared to those with non‐painful or no symptoms, regardless of the OHIP version. The second hypothesis was partly supported, as summary scores across the OHIP versions were strongly or very strongly correlated. The third hypothesis was also partially supported. OHIP‐5 demonstrated measurement coherence with OHIP‐14 and OHIP‐TMD, reflected in strong correlations and consistent differentiation across TMD symptom groups in overall OHRQoL. The OHIP‐5 can be used for general OHRQoL research in TMD patients, but its substitution for OHIP‐14 or OHIP‐TMD is appropriate only when brevity is prioritised over dimension‐specific assessment.

### 4.1 Prevalence of TMD Symptoms

University students were selected as the young adult population due to the high prevalence of TMDs among them and the potential impact of TMDs on academic performance, daily activities, and general well‐being [35, 36]. Furthermore, if the different TMD subtypes can be distinguished using various OHIP versions in nonclinical samples, they are likely even more effective in TMD patient populations given their more pronounced symptoms and functional impairments. The 5Ts screener demonstrates high accuracy in detecting TMDs and has been utilised in both general and clinical population research [30, 36]. Recently, it was expanded to assess the duration, frequency, intensity, and interference of specific TMD symptoms, as well as their overall burden [37]. The 36.0% prevalence of TMD symptoms in this study was consistent with the 37.0%–44.3% reported in other Asian university student samples [38, 39]. IT symptoms were the most frequent, followed by CT and PT symptoms, mirroring the pattern of TMD diagnoses in young adult patients [40].

### 4.2 Comparison of OHIP Versions

Across all OHIP versions, participants with TMD symptoms, especially those with combined symptoms, reported poorer OHRQoL than those without. Despite its markedly reduced number of items, the OHIP‐5 effectively distinguished the impact of various TMD symptoms on overall OHRQoL. Significant differences among participant groups in the Physical Pain (Do2), Social Disability (Do6), and Handicap (Do7) domains were consistent across OHIP‐49, OHIP‐14, and OHIP‐TMD, while other domains exhibited variations. OHIP‐49 and OHIP‐TMD produced similar outcomes for the Functional Limitation (Do1) and Psychological Discomfort (Do3) domains, while both OHIP‐49 and OHIP‐14 differed from OHIP‐TMD in the Physical Disability (Do4) and Psychological Disability (Do5) domains. These findings indicate that the choice of OHIP version can influence the measurement of specific OHRQoL domains, particularly those related to physical and psychological aspects, which are more affected by TMDs [12].

Significant differences among participant groups across all four dimensions were observed using the OHIP‐49, OHIP‐14, OHIP‐5, and OHIP‐TMD. The OHIP‐49 and OHIP‐TMD yielded comparable outcomes for the Orofacial Function (Di1) and Psychosocial Impact (Di4) dimensions, while the OHIP‐14 produced similar results to the OHIP‐TMD for the Orofacial Appearance (Di3). However, differences were identified in the evaluation of Orofacial Pain (Di2) when comparing the OHIP‐49, OHIP‐14, and OHIP‐5 to the OHIP‐TMD, and the OHIP‐5 failed to distinguish between participant groups in the Psychosocial Impact (Di4) dimension. This demonstrates that the condition‐specific OHIP‐TMD may provide a more effective assessment of pain and psychosocial impacts of TMDs compared to generic abbreviated measures, particularly the OHIP‐5.

### 4.3 Correlation Analysis

The OHIP‐49, OHIP‐14, and OHIP‐5 summary scores showed strong associations with OHIP‐TMD, affirming that multiple OHIP versions can effectively assess overall OHRQoL in individuals with TMDs. In both the OHIP‐14 and OHIP‐TMD, the four dimensions exhibited the strongest correlations with the following domains: Orofacial Function (Di1) with Physical Disability (Do4), Orofacial Pain (Di2) with Physical Pain (Do2), Orofacial Appearance (Di3) with Psychological Discomfort (Do3), and Psychosocial Impact (Di4) with Psychological Disability (Do5). These correlations were strong to very strong. The results supported the recommendations of the international team, except for the Psychosocial Impact (Di4) dimension, where the Handicap (Do7) domain was regarded as the most representative [15]. For both the OHIP‐14 and OHIP‐TMD, the association between the Psychosocial Impact (Di4) dimension and the Handicap (Do7) domain was only moderate. Findings suggest that while TMD symptoms affect psycho‐emotional functioning, their impact may not reach the severity of life limitations characterised by the Handicap domain. This aligns with observations in TMD patients, where the Psychological Disability (Do5) domain contributed the majority of the items in the primary component of the OHIP‐TMD [41].

As the four‐dimensional framework and OHIP‐5 could potentially serve as the universal dPROM across all oral conditions, its correlations with the OHIP‐14 and OHIP‐TMD domains were also of interest [15, 27]. Additionally, item 26 was omitted from the secondary analysis due to its limited relevance to TMDs. Several strong relationships were found between the OHIP‐5 dimensions and the domains of the OHIP‐14 and OHIP‐TMD. Orofacial Pain (Di2) of the OHIP‐5 showed a strong correlation with Physical Pain (Do2), while Psychosocial Impact (Di4) was strongly related to Social Disability (Do6) of the OHIP‐14. Furthermore, strong correlations were noted between Orofacial Function (Di1), excluding item 26, of the OHIP‐5 and Functional Limitation (Do1); Orofacial Pain (Di2) and Physical Pain (Do2); and Psychosocial Impact (Di4) and Social Disability (Do6) of the OHIP‐TMD. These associations suggest that the OHIP‐5 dimensions might examine different constructs than the OHIP‐14 and OHIP‐TMD dimensions.

The Orofacial Function (Di1) dimension of the OHIP‐5 was moderately correlated with most OHIP‐14 and OHIP‐TMD domains. Removing item 26 from this dimension resulted in a subtle but consistent decline in correlation strengths, except for the Functional Limitation domain (Do1) of the OHIP‐TMD, where the correlation increased from 0.68 to 0.88. This pattern indicates that item 26 contributes meaningfully to the overall OHRQoL construct by capturing sensory‐related impairments that overlap with broader pain and disability domains. Collectively, the findings determined that the four‐dimensional impact framework is suitable for use in TMD research. While the OHIP‐5 effectively evaluates overall OHRQoL, it may not be appropriate for assessing the multidimensional impact of TMDs, particularly in the Psychosocial Impact dimension, where it failed to differentiate between TMD subtypes.

### 4.4 Study Limitations

The study has several strengths, including its large sample size and the use of both generic and TMD‐specific OHIP versions. Nevertheless, it also faced certain methodological limitations. First, the study sample consisted solely of young adults from a single geographic region and included a higher proportion of women, which may reduce the generalizability of the findings to other age groups, racial backgrounds, and men. Second, although the 5Ts screener has high diagnostic accuracy, its reliance on self‐reported symptoms makes it susceptible to recall, social desirability, and other partialities. Similarly, the OHIP, as a dPRO, is subject to similar information biases [42]. Third, participant group classifications were based on TMD symptoms rather than definitive diagnoses. However, definitive TMD diagnosis requires physical examinations and diagnostic imaging, the latter being impractical for large‐scale, community‐based studies. Fourth, confirmatory factor analysis (CFA) was not performed for the OHIP‐5. Such analysis could test its unidimensionality, support construct validity, and evaluate model fit, thereby strengthening its justification as a summary measure and its comparability across TMD subgroups. Lastly, the cross‐sectional design of the study prevents the evaluation of the causal relationship or the tracking of longitudinal changes between TMD symptoms and OHRQoL. Additionally, the generic OHRQoL measures may have been influenced by other oral conditions such as malocclusion, dental caries, and periodontal disease, which were not evaluated in this study [43]. Future research could adopt a longitudinal design, incorporate a more diverse sample with respect to age, race, and gender, replace the evaluation of subjective TMD symptoms with objective diagnoses, validate the OHIP‐5 against other DC/TMD Axis II instruments such as the Graded Chronic Pain Scale and the Jaw Functional Limitation Scale, and perform CFA to evaluate its dimensional structure and model fit.

## 5 Conclusion

This study revealed that TMD symptoms, particularly those involving pain, were associated with reduced OHRQoL among young adults. The use of a four‐dimensional impact framework in TMD research was validated, and its alignment with established OHIP domains was clarified. While OHIP‐14 and OHIP‐TMD captured dimension‐specific impacts effectively, OHIP‐5 was limited in detecting psychosocial differences, which underscored its role as a global measure rather than a dimensional tool. These results support the targeted application of OHIP instruments based on research aims and highlight the value of the four‐dimensional framework in improving conceptual clarity and clinical relevance.

## Conflicts of Interest

The authors declare no conflicts of interest.

## Data Availability

The data that support the findings of this study are available from the corresponding author upon reasonable request.
